# CAN INTERMITTENT FASTING SLOW PRIMARY AND SECONDARY AGING IN HUMANS?

**DOI:** 10.1093/geroni/igae098.0580

**Published:** 2024-12-31

**Authors:** Courtney Peterson

**Affiliations:** University of Alabama Birmingham, Birmingham, Alabama, United States

## Abstract

Calorie restriction slows primary and secondary aging and increases lifespan in multiple animal species. However, recent evidence suggests that calorie restriction extends lifespan largely via extended fasting periods rather than energy restriction per se. The practice of periodically extending the fasting period is known as intermittent fasting. Dozens of studies in animals suggest that intermittent fasting decreases body weight; reduces the incidence of and/or progression of several chronic diseases; and extends lifespan. In humans, there are a wide range of intermittent fasting approaches, including alternate-day fasting, alternate-day modified fasting, the 5:2 diet, the fasting-mimicking diet, and time-restricted eating (TRE). In this presentation, I will briefly review evidence showing that intermittent fasting extends lifespan in animals. I will then define and discuss the various forms of intermittent fasting studied in humans. I will cover what is known about the feasibility of different types of intermittent fasting and their effects on primary and secondary aging in humans. I will conclude with a deeper dive into the most popular form of intermittent fasting, TRE. I will discuss its effects on weight loss, cardiometabolic health, and primary and secondary aging in humans.

